# Supplementary material for: Pulmonary Fibroelastotic Remodelling Revisited
Source: Cells. 2021 Jun 1;10(6):1362. doi: 10.3390/cells10061362 (PMC8227669; doi:10.3390/cells10061362)
Supplement: Supplementary file 1 [file cells-10-01362-s001.zip › cells-1211948-supplementary.pdf]

Supplementary material for Braubach et al.: “Pulmonary fibroelastotic remodeling revisited”

Supplementary table S1: Detailed patient information:

Detailed clinical information for the investigated patient collective including the group age at time of surgery and sex.

	Group	Age	Sex	Clincial information
1	PAC	75	m	Partial upper lobe resection due to emphysematous bullae. Incidental PAC.
2	PAC	57	m	Upper lobe resection due to adenocarcinoma. Incidental PAC.
3	PAC	71	f	Partial upper lobe resection due to adenocarcinoma. Incidental PAC.
4	PAC	67	f	Upper lobe resection due to squamous cell carcinoma. Incidental PAC.
5	PAC	40	f	Partial upper lobe resection due to metastasis of renal cell carcinoma. Incidental PAC.
6	PAC	69	f	Partial upper lobe resection due to recurrent adenocarcinoma. Incidental PAC.
7	PAC	62	f	Double lung transplantation due to pulmonary hypertension. Incidental PAC.
8	PAC	74	f	Resection of upper lobe due to adenocarcinoma. Incidental PAC.
9	PAC	31	f	Partial upper lobe resection due to recurrent spontaneous pneumothorax. Incidental PAC.
10	PAC	25	m	Partial upper lobe resection due to recurrent spontaneous pneumothorax. Incidental PAC.
11	PAC	63	m	Partial upper lobe resection due to unclear apical mass and suspected cancer metastasis. Histologic analysis revealed PAC as correlate of apical mass.
12	PAC	23	m	Partial upper lobe resection due to recurrent spontaneous pneumothorax. Incidental PAC.
13	PAC	68	f	Partial upper lobe resection due to adenocarcinoma. Incidental PAC.
14	PAC	77	f	Partial upper lobe resection due to apical mass and suspected lung cancer. Histologic analysis revealed PAC as correlate of apical mass.
15	CLAD	54	m	Re-DLTX after 3 years due to CLAD/RAS. Primary diagnosis was pulmonary fibrosis with NSIP pattern.
16	CLAD	59	m	Re-DLTX after 4 years due to CLAD/RAS. Primary diagnosis was pulmonary emphysema without AAT-deficiency.
17	CLAD	48	f	Re-DLTX after 4 years due to CLAD/RAS. Primary diagnosis was pulmonary fibrosis with UIP pattern.
18	CLAD	23	f	Re-DLTX after 2 years due to CLAD/BOS. Primary diagnosis was CF with bronchiectasis.
19	CLAD	35	f	Re-DLTX after 7 years due to CLAD/RAS. Primary diagnosis was CF with bronchiectasis.
20	CLAD	45	f	Re-DLTX after 5 years due to CLAD/BOS. Primary diagnosis was CF with bronchiectasis.
21	CLAD	38	f	Re-DLTX after 10 years due to CLAD/BOS. Primary diagnosis was pulmonary emphysema.
22	CLAD	55	f	Re-DLTX after 14 years due to CLAD/BOS. Primary diagnosis was pulmonary emphysema.
23	CLAD	38	f	Re-DLTX after 5 years due to CLAD/BOS. Primary diagnosis was CF with bronchiectasis.
24	CLAD	50	m	Re-DLTX after 5 years due to CLAD/RAS. Primary diagnosis was pulmonary emphysema.
25	CLAD	44	m	Re-DLTX after 2 years due to CLAD/RAS. Primary diagnosis was pulmonary fibrosis with UIP pattern and on the basis of an exogenic allergic alveolitis.

26	CLAD	51	f	Re-DLTX after 8 years due to CLAD/BOS. Primary diagnosis was pulmonary veno-occlusive disease.
27	GvHD	24	m	Partial upper lobe resection due to pneumonia and bronchiectasis. HSCT due to ALL.
28	GvHD	15	f	DLTX due to HSCT associated fibrosis/BO 10 years after HSCT due to leukemia.
29	GvHD	57	f	DLTX due to HSCT associated fibrosis/BO 19 years after HSCT due to bilinear NHL.
30	GvHD	15	m	DLTX due to BO and recurrent pneumothorax 6 years after HSCT due to bcr/abl positive leukemia.
31	GvHD	20	m	DLTX due to HSCT associated fibrosis/BO 2 years after HSCT due HL.
32	GvHD	32	m	DLTX due to BO and recurrent pneumothorax 3 years after HSCT due to bcr/abl positive leukemia.
33	GvHD	9	m	DLTX due to BO and recurrent pneumothorax 3 years after HSCT due to combined immunodeficiency.
34	ILD	49	f	DLTX due to pulmonary fibrosis (primary diagnosis was 3 years before) with focal UIP pattern and concomitant AFE pattern. Clinically suspected exogenic allergic alveolitis.
35	ILD	47	f	DLTX due to pulmonary fibrosis in stage IV sarcoidosis (primary diagnosis was made 19 previously) interstitial fibrosis and concomitant AFE pattern.
36	iPPFE	54	f	DLTX due to interstitial fibrosis with clinical and radiological PPFE pattern and no determinable cause. Primary diagnosis was made 2 years before.
37	RCTX	52	f	DLTX due to interstitial fibrosis after radio-chemotherapy of HL. Primary diagnosis of pulmonary fibrosis was made 1 year before.
38	iPPFE	58	f	DLTX due to interstitial fibrosis with clinical and radiological PPFE pattern and no determinable cause. Primary diagnosis was made 2 years before.
39	iPPFE	61	m	DLTX due to interstitial lung disease with radiological UIP pattern and no known cause. Histologic examination revealed wide-spread AFE pattern lung fibrosis. Primary diagnosis was made 2 years before.
40	AID	45	f	DLTX due to pulmonary fibrosis on the basis of rheumatoid arthritis. Primary diagnosis was made 11 years before.
41	iPPFE	56	f	DLTX due to interstitial fibrosis with clinical PPFE pattern and no determinable cause. Primary diagnosis was made 2 years before
42	AID	44	m	DLTX due to pulmonary fibrosis and hypertension on the basis of a mixed connective tissue disease. Primary diagnosis was made 35 years before.
43	RCTX	58	f	Partial lung resection due to suspected recurrent adenocarcinoma treated with radiotherapy 1 year before. Widespread AFE pattern fibrosis upon histological examination.
44	iPPFE	59	m	DLTX due to interstitial fibrosis with clinical and radiological PPFE pattern and no determinable cause.
45	iPPFE	24	m	DLTX due to suspected histiocytosis. Histological examination showed wide-spread AFE patter pulmonary fibrosis.

Abbreviations

AAT	Alpha-1-Antitrypsin
ALL	Acute lymphocytic leukemia
CF	Cystic fibrosis
CLAD	Chronic lung allograft dysfunction
DLTX	Double lung transplantation
f	Female
HL	Hodgkin lymphoma
LTX	Lung transplantation
m	Male
NHL	Non-Hodgkin lymphoma
NSIP	Nonspecific interstitial pneumonia
PAC	Pulmonary apical cap
RAS	Restrictive allograft syndrome
UIP	Usual interstitial pneumonia

Supplementary figure S1

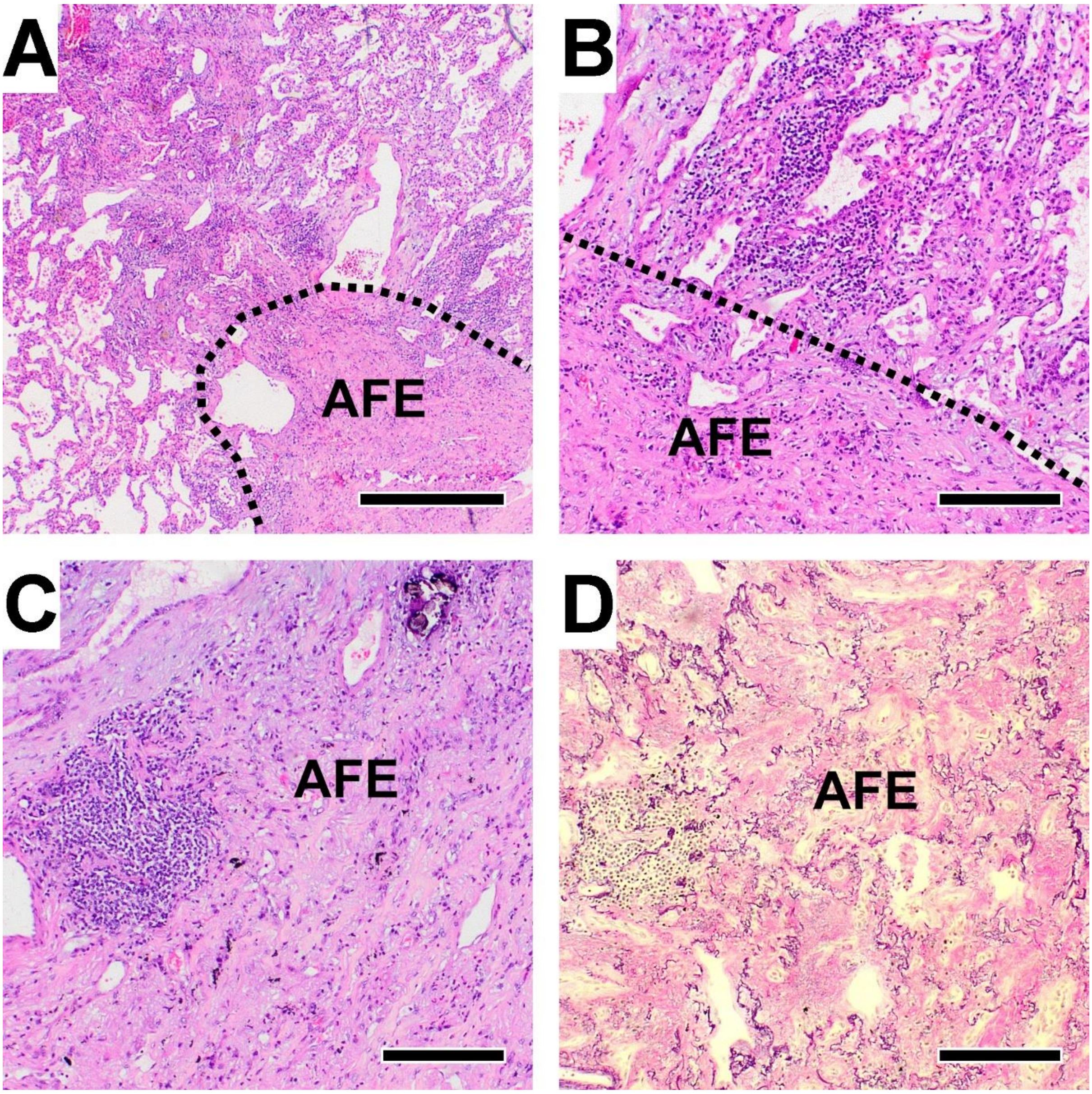

Supplementary figure S1: Alveolar fibroelastosis (AFE) in concomitant interstitial lung disease (ILD): AFE can present as secondary pattern in pulmonary fibrosis with other patterns (e.g. usual interstitial pneumonia). In this case (Case no 34) regions with AFE-pattern fibrosis show little to no (diffuse) lymphocytic infiltrate. In contrast remodeled parenchyma without AFE shows marked lymphocytic infiltration (A, B). In regions with AFE occasional lymphocytic aggregates, mostly in the periphery of the lesion, can be observed (C, D). A-C are stained with hematoxylin and eosin, D with elastic van Giesson. Scalebars are A: 600 µm, B-D: 200 µm.
